# Impacts of wind stilling on solar radiation variability in China

**DOI:** 10.1038/srep15135

**Published:** 2015-10-14

**Authors:** Changgui Lin, Kun Yang, Jianping Huang, Wenjun Tang, Jun Qin, Xiaolei Niu, Yingying Chen, Deliang Chen, Ning Lu, Rong Fu

**Affiliations:** 1 Key Laboratory of Tibetan Environment Changes and Land Surface Processes, Institute of Tibetan Plateau Research, Chinese Academy of Sciences, Beijing, China; 2 CAS Center for Excellence in Tibetan Plateau Earth System Sciences, Chinese Academy of Sciences, Beijing, China; 3 College of Atmospheric Sciences, Lanzhou University, Lanzhou, China; 4 Department of Earth Sciences, University of Gothenburg, Gothenburg, Sweden; 5 State Key Laboratory of Resources and Environmental Information System, Institute of Geographic Sciences and Natural Resources Research, Chinese Academy of Sciences, Beijing, China; 6 Department of Geological Sciences, Jackson School of Geosciences, the University of Texas at Austin, Austin, Texas, USA

## Abstract

Solar dimming and wind stilling (slowdown) are two outstanding climate changes occurred in China over the last four decades. The wind stilling may have suppressed the dispersion of aerosols and amplified the impact of aerosol emission on solar dimming. However, there is a lack of long-term aerosol monitoring and associated study in China to confirm this hypothesis. Here, long-term meteorological data at weather stations combined with short-term aerosol data were used to assess this hypothesis. It was found that surface solar radiation (SSR) decreased considerably with wind stilling in heavily polluted regions at a daily scale, indicating that wind stilling can considerably amplify the aerosol extinction effect on SSR. A threshold value of 3.5 m/s for wind speed is required to effectively reduce aerosols concentration. From this SSR dependence on wind speed, we further derived proxies to quantify aerosol emission and wind stilling amplification effects on SSR variations at a decadal scale. The results show that aerosol emission accounted for approximately 20% of the typical solar dimming in China, which was amplified by approximately 20% by wind stilling.

The solar lights up our sky and, as the ultimate energy source, it drives water, energy and carbon cycles on the Earth surface. Cloudiness, aerosols and water vapor modulate the transparency of atmosphere and alter the solar radiation incident at the Earth surface (referred to as surface solar radiation, SSR), and may therefore yield profound climatic, environmental, and socioeconomic consequences^1^,^2^,^3^. A pronounced transition “from solar dimming to brightening” circa 1990 in many regions of the world was reported based on the evidence from ground measurements and satellite observations^4^,^5^,^6^, which tends to be attributed to the changes in mass and properties of aerosols. In China, however, recent studies^7^,^8^ employing quality controlled SSR data have reported a leveling-off rather than brightening of solar radiation over the dimming period. Increasing aerosol concentrations were considered as the major contributor to the solar dimming^9^,^10^,^11^ and a plausible explanation of the absence of solar brightening over China^12^. In addition, wind blowing may increase the rate of aerosol dispersion and modulate SSR^13^. During the last decades, widespread wind stilling has occurred over the Northern Hemisphere^14^ including China^15^,^16^, which is consistent with the increased aerosol loading and SSR changes; therefore, wind stilling may have amplified the aerosol effect on solar dimming. This effect was assessed by ref. 13, which included an evaluation of the air pollution index (API) data measured in 27 cities of China. However, API does not indicate the concentration of fine atmospheric particles (e.g. PM_2.5_ defined with aerodynamic diameters ≤2.5 μm) that is closely related with aerosol optical depth (AOD), as fine particles with a size comparable to the wavelength of visible light can efficiently attenuate solar radiation^17^,^18^,^19^. Moreover, direct measurements of the aerosol loads in China over the past half-century are not available, which makes it difficult to prove the above-mentioned hypotheses on the aerosol and wind stilling effects.

We circumvented the lack of long-term AOD measurements and examined the wind-aerosol-SSR hypothesis from historic routine weather station data in China. In turn we derived aerosol effects on SSR, which were used to correlate the individual contributions of aerosol emission and wind stilling with SSR variations over the last decades in China.

## Observed SSR changes and wind stilling

**Figure 1:**
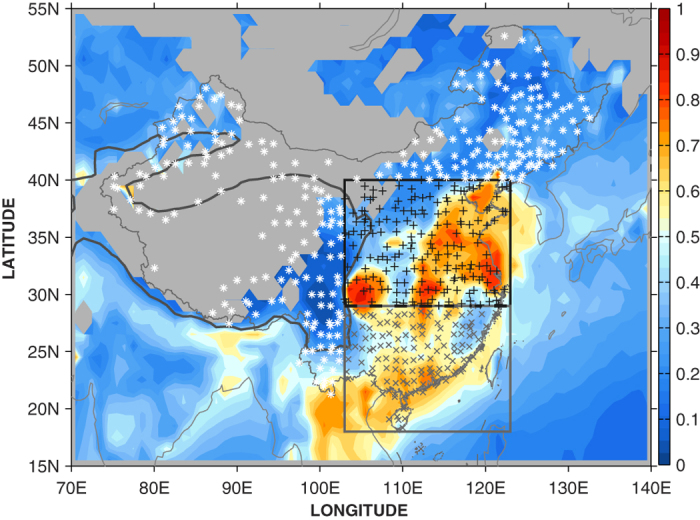
The distribution of CMA stations (markers) and the spatial pattern of winter half-year climatology of MODIS AOD (colors) in mainland of China. Three sub-regions are defined: black “+” (inside the black box) as the region of central-eastern China (CE); grey “x” (inside the grey box) as the region of southern China (SC); and white “*” (outside the two boxes) as the “other” (OT) region. The grey background without colors covered indicates missing AOD values. The map was created using MATLAB.

To clarify the role of aerosol effects on SSR variations, three sub-regions with different pollution densities have been defined according to the distribution of AOD values (see Fig. 1). The sub-regions include the heavily polluted central-eastern region of China (CE), the moderately polluted southern region of China (SC), and slightly polluted other regions of China (OT).

The decadal SSR variations in the three sub-regions were calculated with quality-controlled SSR data^20^ (see Supplementary Figure S1a). During the “dimming” period (1970–1989), the most substantial decrease in SSR occurred in CE and SC (−4.4 ± 0.5 and −4.3 ± 0.6 W m^−2^ decade^−1^, respectively), and the dimming magnitude in OT was relatively small (−1.9 ± 0.1 W m^−2^ decade^−1^). The solar brightening since 1990 was only observed in SC (1.6 ± 0.5 W m^−2^ decade^−1^). However, the SSR in CE continued dimming after 1990, although the dimming rate was rather weak (−0.4 ± 0.4 W m^−2^ decade^−1^) compared with that of the “dimming” period. Throughout China, subsequent to the dimming period was a leveling-off period of SSR beginning in 1990, although earlier studies^21^,^22^,^23^ reported that a brightening period began instead.

Wind stilling is another outstanding climate change that has occurred in China^15^,^16^ since the beginning of the 1970s (see Supplementary Figure S2b). The declining trend in surface wind speed (hereafter refer to as *U*) was alleviated starting in 1990 (−0.07 ± 0.01 m s^−1^ decade^−1^ for the “brightening” period compared with −0.24 ± 0.01 m s^−1^ decade^−1^ for the “dimming” period).

## Decreasing SSR with wind stilling

**Figure 2:**
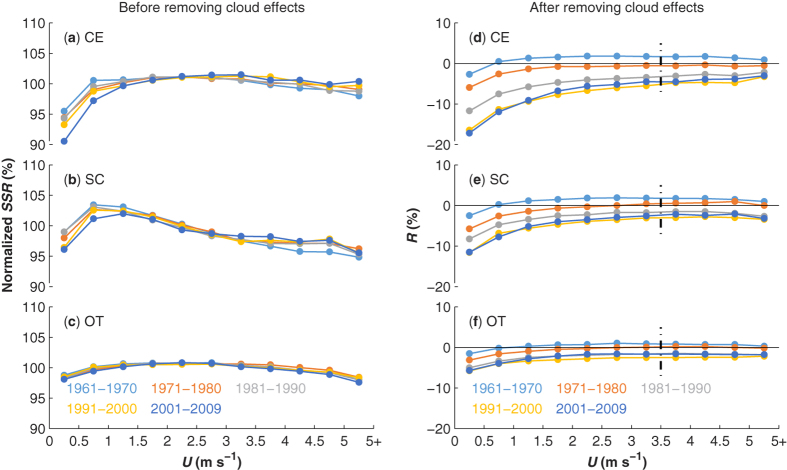
Statistical decadal relationship (a–c) between normalized SSR (daily value relative to monthly mean) and *U* (bins with 0.5 m s^−1^ width) and (d–f) between *R* (the relative residual from cloud effects model of the observed SSR to monthly mean) and *U* for the three individual regions (CE, SC, and OT). Different colors denote the five decades as illustrated at the bottom. The dot-dash lines denote the threshold value of *U*.

Strong wind can effectively disperse aerosols and increase SSR over locally^13^. Thus, SSR along with different levels of *U* strength may alter the aerosol extinction effect on solar radiation. Figure 2a–c shows the decadal relationship between mean values of the daily SSR normalized by monthly mean and the different levels of *U* for the three sub-regions in each specific decade. In CE, the logarithmic-like curves indicate that strong *U* enhances SSR in the heavily polluted region, and the inter-dependence between SSR and *U* is reduced in the relatively clean region (OT), which was expected. However, the dependence between SSR and *U* is not observed in the moderately polluted SC region, which may have been caused by the impact of clouds on SSR. Note that this effect is not excluded in the results presented in Fig. 2a–c.

After removing the cloud effects (refer to the Methods section for additional details), a logarithmic-like relationship is observed between a non-dimensional index of SSR (*R*) and *U* for all the three sub-regions (Fig. 2d–f). The dependence of *R* on *U* is much stronger in the heavily polluted CE region than in the slightly polluted OT region and more clearly demonstrated in recent decades. This result is consistent with the transport and dispersion of aerosols by strong *U*, which enhances SSR. Ref. 13 reports that winds noticeably enhance SSR when *U* < 2.5 m s^−1^ but attenuate SSR when *U* > 3.5 m s^−1^. This enhancement is confirmed here, but the attenuation when *U* > 3.5 m s^−1^ is not seen. Instead, it can be seen in Fig. 2d–f that *R* no longer changes when *U* > 3.5 m s^−1^. This difference may be due to the contamination of cloud effects not excluded in ref. 13. Therefore, we suggest that atmospheric aerosols can be effectively dispersed when *U* is greater than 3.5 m s^−1^ and this value can be considered as a threshold of *U* (*U*_*th*_) to identify clean skies. This effect is also substantiated by the relationship between *U* and PM_2.5_ concentration measured in Beijing (see Supplementary Fig. S2a), a city that suffers from frequent haze. The negative-exponential fitting (using a robust regression with the least absolute residual algorithm^24^) illustrates that stronger *U* values indicate lower PM_2.5_ concentrations. We converted the PM_2.5_ concentrations to individual air quality index (AQI) grades according to the standards of the United States Environmental Protection Agency (USEPA), and then averaged the *U* values for six AQI grades. The averaged *U* is approximately 3.5 m s^−1^, the threshold value for wind speed *U*_*th*_ defined above, when the air quality condition is “good” (see Supplementary Fig. S2b). Again, it indicates that atmospheric aerosols can be effectively dispersed when *U* > *U*_*th*_. Therefore, the offset (deviation of *R* to zero) for *U* > *U*_*th*_ implies errors in estimating decadal cloud effects on SSR because cloud properties can present decadal changes.

The curvature of the *R* curve for *U* < *U*_*th*_ is conspicuous for recent decades and the heavily polluted region (CE) with high AOD values. The difference of *R* between conditions of calm wind and *U*_*th*_ can be as high as 15% in CE (Fig. 2d), indicating that calm weather with high AOD can considerably decrease SSR. Air pollution makes it a compelling hypothesis that wind stilling darkened China’s sky.

## Wind stilling impact on SSR decadal variability

In term of the decadal SSR variability, the *direct aerosol effects* on SSR changes may include the *aerosol emission effect* and the *wind stilling amplification effect*. According to the SSR dependence on *U*, three proxies have been designed for the total direct aerosol effects (*R*_*a*+*u*_), aerosol emission effect (*R*_*a*_) and wind stilling amplification effect (*R*_*u*_) (see the Method section for additional details). The three proxies are calculated based on a 5-year moving window.

**Figure 3:**
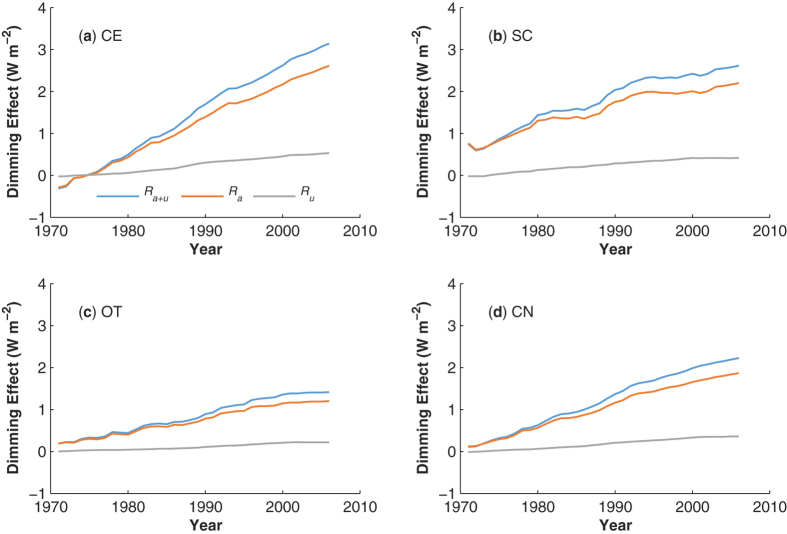
Temporal variations of annual mean direct aerosol effects (blue; *R*_a+u_), aerosol emission effect (orange; *R*_*a*_) and wind stilling amplification effect (grey; *R*_*u*_) on SSR averaged over the three individual regions: (CE, SC, and OT) and throughout China (CN). Ploted values are relative to those before 1970.

Figure 3 illustrates the changes in the three proxies over different regions relative to their values before 1970. The proxies exhibit positive trends that are consistent with the increase of haze days^25^. The contribution of direct aerosol effects to SSR changes is most substantial in the heavily polluted CE region, which is consistent with high the AOD values (Fig. 1) and high API in this region^26^. The wind stilling effect suppressed the solar brightening in China, but this effect only constitutes approximately 20% of the total direct aerosol effects (*R*_*a*+*u*_). Similarly, the most substantial dimming trend contributed by wind stilling occurred in CE, indicating that the role of wind stilling in decadal SSR changes is dependent on the level of pollution.

**Figure 4:**
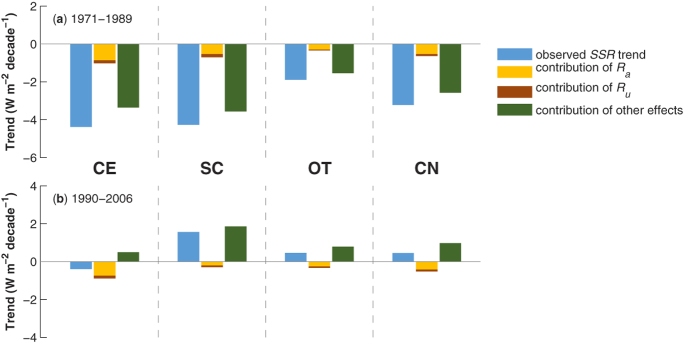
Observed SSR trends and the contribution of *R*_*a*+*u*_ (*R*_*a*_ + *R*_*u*_) and other effects (observed SSR trend – the contribution of *R*_*a*+*u*_) for (**a**) the “dimming” period (1971–1989) and (**b**) the “brightening” period (1990–2006) over the regions of CE, SC, OT, and throughout China (CN).

Figure 4 shows the observed SSR trends and contribution of the direct aerosol effects for the “dimming” period (1971–1989) and “brightening” period (1990–2006). During the “dimming” period, substantial dimming trend of SSR is observed in all regions. The direct aerosol effects (−1.02 ± 0.02, −0.70 ± 0.04, and −0.35 ± 0.01 W m^−2^ decade^−1^ for CE, SC, and OT, respectively) account for about 20% of the dimming trend for the three sub-regions. The wind stilling amplification effect is a small part (about 20%) of the total direct aerosol effects, and its contribution to the dimming is only a few percent. During the “brightening” period, SSR decreased in CE (−0.4 ± 0.4 W m^−2^ decade^−1^) while increased in SC (1.6 ± 0.5 W m^−2^ decade^−1^) (see Supplementary Fig. S1). The direct aerosol effects caused a dimming trend of −0.89 ± 0.02 W m^−2^ decade^−1^ in CE and −0.30 ± 0.03 W m^−2^ decade^−1^ in SC during this period, and of which the wind stilling amplification effect contributes to 20–30% of the direct aerosol effects. The results for the mainland of China is similar to individual regions, i.e., the total direct aerosol effects account for about 20% of solar dimming during the “dimming” period and the wind stilling amplification effect constituted approximately 20% of the total direct aerosol effects. We speculate the remaining part of the SSR variability is due to cloud variability, but we are reluctant to present it, as we do not have sufficient evidences for the long-term change in cloud properties.

We also analyzed the aerosol effects on the decadal variations of SSR in individual months (see Supplementary Fig. S3). Winter is the most polluted season in China, and high API^26^ and haze days^27^ occurred frequently in winter. As expected, the wintertime aerosol effects are the largest among the four seasons and cause a solar dimming equal to 1.5% of the mean SSR for the entire period assessed in this study. This result is consistent with the substantial decline of SSR in winter^22^. In other calendar months, the values of the dimming effect are all lower and/or equal to 1% of the mean SSR.

## Implications

Wind stilling can considerably amplify solar extinction by aerosols at a daily scale. This effect in China can reduce SSR by up to 15% for heavily polluted regions and seasons. The threshold *U* required to effectively remove the aerosol effect is approximately 3.5 m s^−1^, which can be a useful reference for managing industrial activities and vehicles emissions in large cities, such as Beijing. In terms of decadal SSR variability, this effect together with the aerosol emission effect only accounts for approximately 20% of the observed dimming and did not dominate the SSR changes over China. It is important to note that only the direct aerosol effects are taken account in the analysis. The indirect aerosol effects, of which aerosols serve as cloud condensation nuclei, can produce additional solar dimming^28^. Thus, with potential wind speed reversals^16^ and strict control on aerosol emission, the current solar dimming may be alleviated and polluted regions may experience brightening in the future.

### Estimation of cloud effects on SSR

SSR variations are associated with TCC and LCC (low-level cloud cover) changes over China^29^,^30^. To construct a proxy for aerosol effects, cloud effects should first be removed from the SSR record. We used TCC and LCC data to construct a quadratic model for estimating the cloud effects on SSR. The quadratic model is specified as follows:


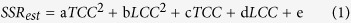


where *SSR*_*est*_ is the daily estimated SSR, and the coefficients (a, b, c, d, and e) are calibrated at each CMA station using the daily SSR, TCC, and LCC of the 1960s, when aerosol emissions were relatively low^27^. It should be noted that Equation (1) is constructed for each calendar month using daily data so that the impacts of seasonal changes in solar zenith angle, water vapor, ozone, and aerosol emission climatology over the 1960s have been implied in the coefficients of this equation. This model cannot be directly used to detect cloud effects on SSR decadal variability, because the model does not account for the impacts of changes in (1) cloud properties, (2) water vapor and ozone, and (3) aerosol concentration. As it is not expected that the first two terms depend on wind speed at a decadal scale, the relationship between the SSR difference (observation minus modeling) and wind speed is an indicator of aerosol effects. We finally define a non-dimensional index (*R*, %) of SSR with cloud effects excluded, using the following equation:


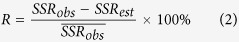


where *SSR*_*obs*_ is daily observed SSR, *SSR*_*est*_ the daily estimated SSR, and 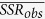 the monthly mean value of the observed SSR.

### Proxies for aerosol effects on SSR

A long-term record of aerosol loads is typically required to quantify the contribution of aerosol effects on decadal SSR variations. Visibility is often used as a proxy of AOD in China but it mainly contains information in the horizontal direction. Here, we used the dependence of SSR on wind speed after removing the cloud effects, which is shown in Fig. 2d–f, to construct proxies for aerosol direct effects on SSR by integrating the SSR and *U* information as follows:


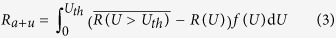


where *R*_*a*+*u*_ (%) is the proxy for the direct aerosol effects on SSR, *U*_*th*_ the threshold of *U*, and *f* () is the probability density function (pdf).

The proxies were calculated for each month in a 5-year moving window at each weather station in China. To separate the impact of aerosol emission and wind stilling, the value of *f* (*U*) in Equation (2) must be obtained for a reference period that is not sensitive to the separation. Here, we used the 1960s as the reference period. The proxy for aerosol emission effect (*R*_*a*_) is calculated as follows:


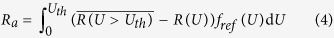


where *f*_*ref*_ () is the reference pdf over the period from 1961–1970.

The proxy for wind stilling amplification effect (*R*_*u*_) can be calculated as follows:


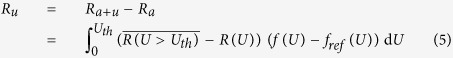


### Data

The quality-controlled^20^ daily SSR dataset (available at http://dam.itpcas.ac.cn) used in the study covers the period from 1961–2010 and includes 716 China Meteorological Administration (CMA) stations. Daily data of *U* (10 m height), TCC, LCC, and precipitation for the period from 1961–2009 at 629 CMA stations are from the China Meteorological Data Sharing Service System at http://old-cdc.cma.gov.cn/, which is governed by the CMA. A total of 573 stations (Fig. 1) that have data of SSR and all the meteorological parameters covering the entire period from 1961–2009. Only days with no precipitation were analyzed because precipitation can significantly reduce the aerosol concentration through wet deposition^31^,^32^.

PM_2.5_ data at 7 monitoring sites in the Beijing urban area are from the China National Environmental Monitoring Center, which is managed by China’s Ministry of Environmental Protection. This dataset includes 184 days of measurements (from 1st March to 31st August, 2013), of which 146 days have no precipitation. It should be noted that China did not measure routine PM_2.5_ until 2012. The monthly AOD data were provided by a Moderate-resolution Imaging Spectroradiometer (MODIS) product (version 5.1, with 1° resolution) and is accessible via the Earth Observing System Data and Information System (EOSDIS) of the National Aeronautics and Space Administration (NASA).

## Additional Information

**How to cite this article**: Lin, C. *et al.* Impacts of wind stilling on solar radiation variability in China. *Sci. Rep.* **5**, 15135; doi: 10.1038/srep15135 (2015).

## Supplementary Material

Supplementary Information
