# Supplementary material for: Impacts of wind stilling on solar radiation variability in China
Source: Sci Rep. 2015 Oct 14;5:15135. doi: 10.1038/srep15135 (PMC4604519; doi:10.1038/srep15135)
Supplement: Supplementary Information [file srep15135-s1.pdf]

Supplementary figures of "Impacts of wind stilling on solar radiation variability in China"

Changgui Lin[1,2], Kun Yang[1,2,*], Jianping Huang[3], Wenjun Tang[1,2], Jun Qin[1], Xiaolei Niu[1,2], Yingying Chen[1,2], Ning Lu[4], Rong Fu[5], and Deliang Chen[6]

[1] Key Laboratory of Tibetan Environment Changes and Land Surface Processes, Institute of Tibetan Plateau Research, Chinese Academy of Sciences, Beijing, China.

[2] CAS Center for Excellence in Tibetan Plateau Earth System Sciences, Chinese Academy of Sciences, Beijing, China.

[3] College of Atmospheric Sciences, Lanzhou University, Lanzhou, China.

[4] State Key Laboratory of Resources and Environmental Information System, Institute of Geographic Sciences and Natural Resources Research, Chinese Academy of Sciences, Beijing, China.

[5] Department of Geological Sciences, Jackson School of Geosciences, the University of Texas at Austin, Austin, Texas, U.S.A.

[6] Department of Earth Sciences, University of Gothenburg, Gothenburg, Sweden.

Supplemental Figures

Fig. S1. Temporal variations of (**a**) anomalies of the annual mean SSR (SSR') and (**b**) anomalies of the annual mean U (U') after 5-yr moving smoothing in the regions of CE, SC, OT and throughout China (CN). Straight lines show the linear trends of SSR in CE and SC for the period of 1990-2006.

Fig. S2. (**a**) PM2.5 concentration vs. U and (**b**) mean value and standard error of U for each AQI grade. Different colors are corresponding to USEPA's air quality graduations: green as "good"; yellow as "moderate"; orange as "unhealthy for sensitive groups"; red as "unhealthy"; purple as "very unhealthy"; and maroon as "hazardous". The exponential fitting for $PM_{2.5}$ concentration on U is using the robust regression with the least absolute residual (LAR) algorithm.

Fig. S3. Temporal variations of (**a**) the direct aerosol effects (R_{a+u}), (**b**) the aerosol emission effect (R_a), and (**c**) the wind stilling amplification effect (R_u) on SSR averaged over all the CMA stations. Plots are relative to their values before 1970. Number denotes each of the twelve calendar months (lines with different colors).

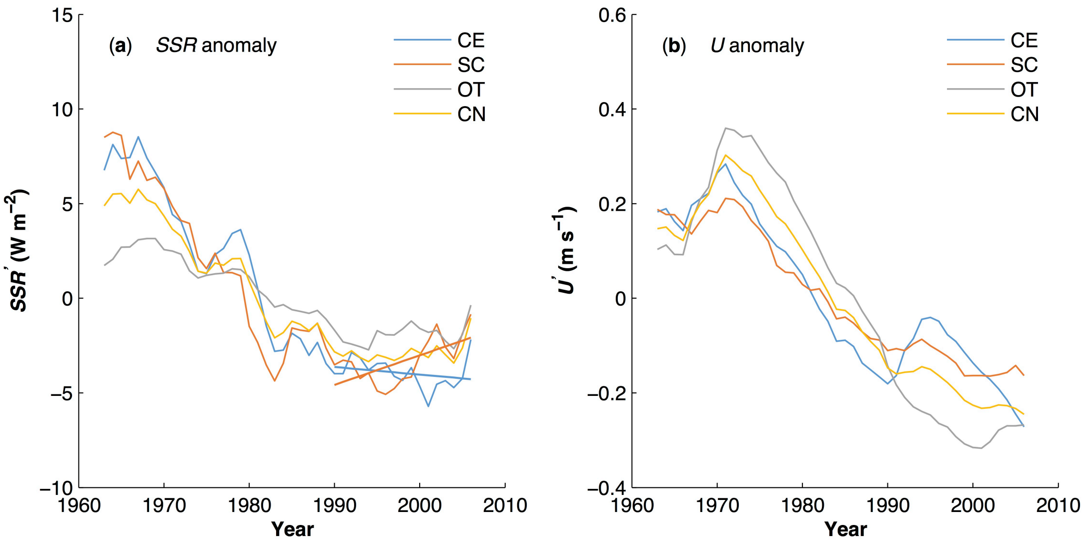

Fig. S1. Temporal variations of (**a**) anomalies of the annual mean SSR (SSR') and (**b**) anomalies of the annual mean *U* (*U*') after 5-yr moving smoothing in the regions of CE, SC, OT and throughout China (CN). Straight lines show the linear trends of SSR in CE and SC for the period of 1990−2006.

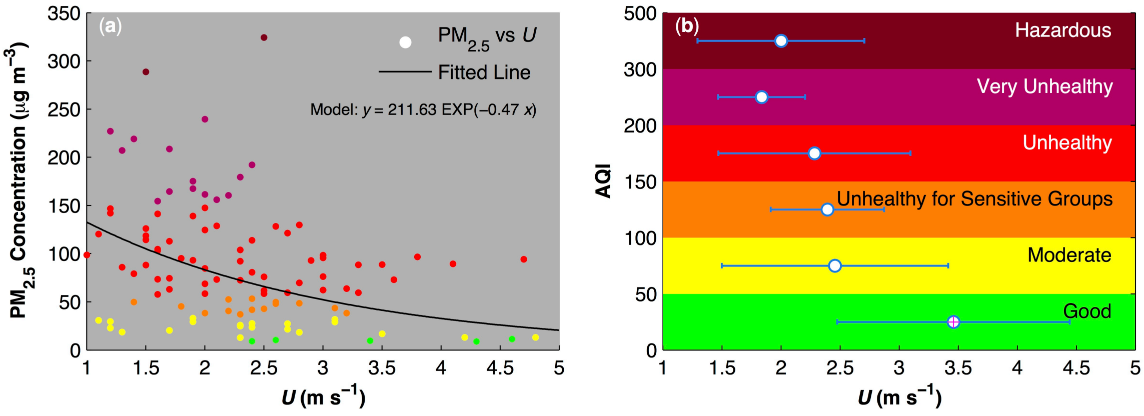

Fig. S2. (**a**) PM2.5 concentration vs. *U* and (**b**) mean value and standard error of *U* for each AQI grade. Different colors are corresponding to USEPA's air quality graduations: green as "good"; yellow as "moderate"; orange as "unhealthy for sensitive groups"; red as "unhealthy"; purple as "very unhealthy"; and maroon as "hazardous". The exponential fitting for $PM_{2.5}$ concentration on *U* is using the robust regression with the least absolute residual (LAR) algorithm.

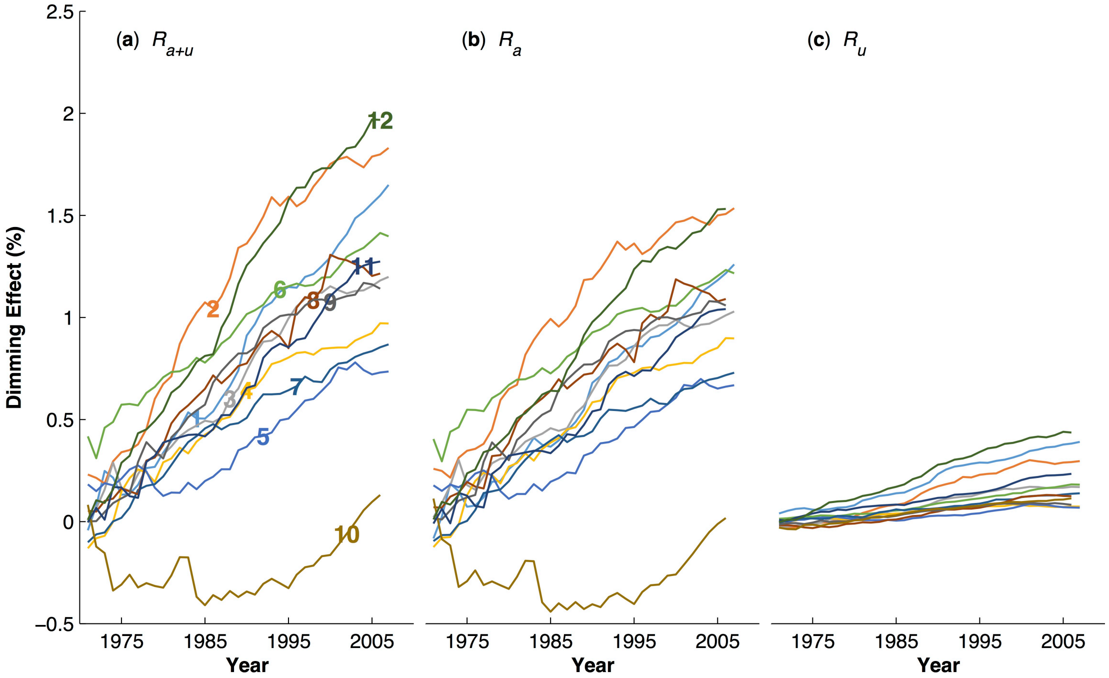

Fig. S3. Temporal variations of (**a**) the direct aerosol effects (R_{a+u}), (**b**) the aerosol emission effect (R_a), and (**c**) the wind stilling amplification effect (R_u) on SSR averaged over all the CMA stations. Plots are relative to their values before 1970. Number denotes each of the twelve calendar months (lines with different colors).
